# Adenocarcinoma at Anastomotic Site of Ureterosigmoidostomy Potentially of Urothelial Origin Spreading to the Upper Urinary Tract

**DOI:** 10.1155/2015/564082

**Published:** 2015-11-15

**Authors:** Katsuhiro Makino, Haruki Kume, Teppei Morikawa, Aya Niimi, Tetsuya Fujimura, Tohru Nakagawa, Hiroshi Fukuhara, Yukio Homma

**Affiliations:** ^1^Department of Urology, Graduate School of Medicine, The University of Tokyo, 7-3-1 Hongo, Bunkyo, Tokyo 1138655, Japan; ^2^Department of Pathology, Graduate School of Medicine, The University of Tokyo, 7-3-1 Hongo, Bunkyo, Tokyo 1138655, Japan

## Abstract

Ureterosigmoidostomy is associated with the risk of several late complications including cancer development at anastomotic sites. We present an unusual case with adenocarcinoma of the anastomotic site associated with multiple adenocarcinoma lesions in the upper urinary tract. A 69-year-old man complained of persistent melena and hematuria. He had undergone radical cystectomy for high-grade bladder cancer and ureterosigmoidostomy 30 years before. Colonoscopy showed a tumor at the right ureterocolonic anastomosis, which was endoscopically resected and histologically diagnosed as adenocarcinoma. Seven years later, a tumor of the left ureterocolonic anastomosis associated with hydronephrosis was found. He underwent temporal percutaneous nephrostomy followed by sigmoidectomy and left ureterocutaneostomy. Eighteen months after the operation, computed tomography (CT) detected left renal pelvic tumor with a mass along the former nephrostomy tract. Left nephroureterectomy and resection of the nephrostomy tract tumor revealed adenocarcinoma with multiple lesions of adenocarcinoma in the ureter. These tumors showed atypical immunohistochemistry as a colonic adenocarcinoma: positive for cytokeratin 7, negative for cytokeratin 20, and negative for *β*-catenin nuclear accumulation. Anastomotic site adenocarcinoma of the present case is potentially of urothelial origin because of unusual clinical manifestation and immunohistochemistry as a colon cancer.

## 1. Introduction

Ureterosigmoidostomy is associated with an increased risk of several late complications. Of particular, the incidence of colon adenocarcinoma is 100- to 550-fold higher compared to that of the general population [1]. Here, we report a case with anastomotic site adenocarcinoma which showed unusual clinical manifestation and immunohistochemistry.

## 2. Case Report

A 69-year-old man visited our hospital because of persistent melena and hematuria. Thirty years before, he had undergone radical cystectomy with bilateral ureterosigmoidostomy for high-grade urothelial carcinoma of the urinary bladder. Colonoscopy showed a tumor at the site of the right ureter-colon anastomosis, which was managed by endoscopic mucosal resection and histologically confirmed as adenocarcinoma. He had no known genetic predisposition for colorectal and urothelial carcinoma.

**Figure 1:**
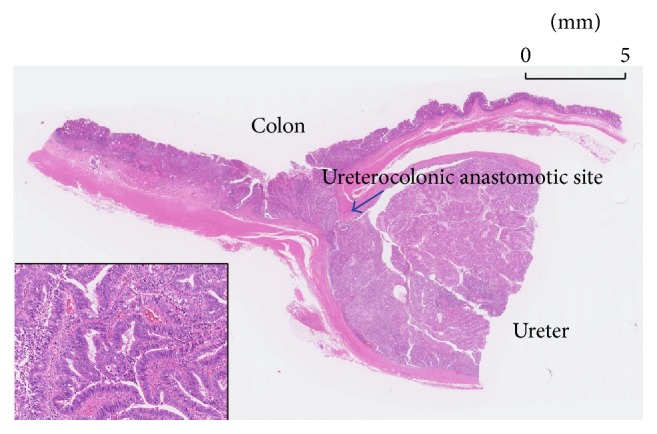
Histology of the left anastomotic site. The tumor spread around the sigmoid colon and ureter, which made the origin of tumor undetermined.

Seven years later, follow-up colonoscopy detected a tumor of the left ureterocolonic anastomosis. Computed tomography (CT) demonstrated invasive colonic cancer and left hydronephrosis. He underwent temporal left percutaneous nephrostomy followed by sigmoidectomy and left ureterocutaneostomy. The tumor was located around the anastomotic site, and it was undetermined whether the tumor had originated from either the colon or the ureter (Figure 1).

**Figure 2:**
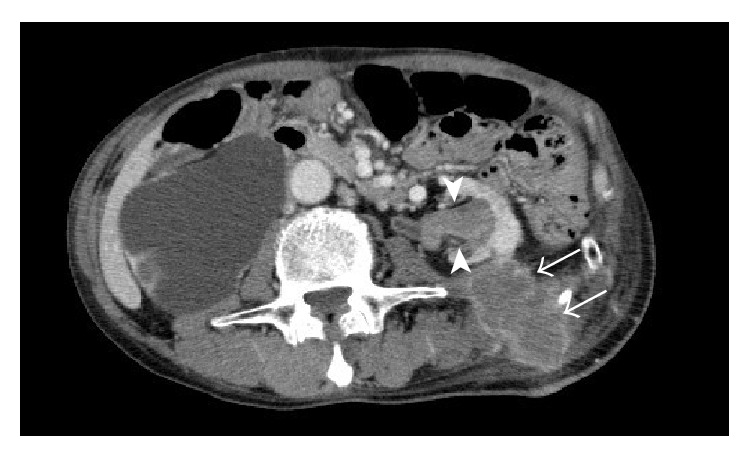
Computed tomography (CT) of the tumors. Abdominal CT scan showing a tumor in the left pelvis (arrowhead) and a soft tissue mass along the former nephrostomy tract (arrow).

**Figure 3:**
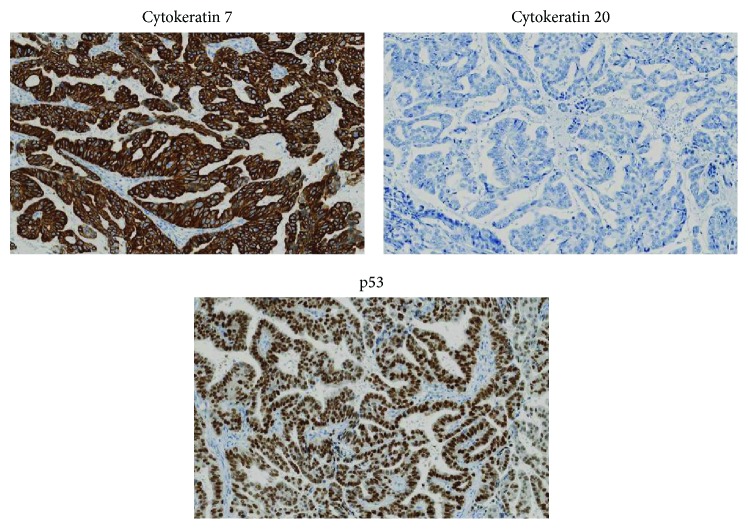
Immunohistochemistry of the anastomosis cancer. Immunohistochemistry demonstrated that these tumors were atypical as a colonic adenocarcinoma: positive for cytokeratin 7, negative for cytokeratin 20, and positive for p53.

Eighteen months after the operation, the patient complained of pain and induration in his left back. CT showed a 2 cm mass in the left renal pelvis and a soft tissue mass posterior to the kidney along the former nephrostomy tract without distant metastasis (Figure 2). We performed left nephroureterectomy and resection of the nephrostomy tract tumor with reconstructive latissimus dorsi musculocutaneous flap. Both the renal pelvic tumor and nephrostomy tract tumor were diagnosed as adenocarcinoma. The ureter was also involved by adenocarcinoma as multiple exophytic lesions as well as adenocarcinoma in situ. The tumors of both ureterocolonic anastomotic sites, the left renal pelvis, nephrostomy tract, and the left ureter were of indistinguishable histological appearance. All these tumors showed a similar immunophenotype: positive for cytokeratin 7, negative for cytokeratin 20, partially positive for CDX-2, positive for p53, and negative for *β*-catenin nuclear accumulation (Figure 3). Three months later, metastasis in lung, paraaortic nodes, and local recurrence were identified on CT. The patient died six months after the operation.

## 3. Discussion

The pathogenesis of adenocarcinoma at the ureterosigmoid anastomotic site is not well understood. A possible mechanism is that the bacterial flora in the colon actively reduced urinary nitrate to nitrite, resulting in formation of carcinogenic N-nitrosocompounds [2–4]. Other possible mechanisms of carcinogenesis include surgical and mechanical trauma, excess concentrations of electrolytes, chronic irritation, and exposure to free radicals for a long period [1]. Apart from carcinogenesis, adenocarcinoma at the anastomotic site is commonly believed to be of colonic origin [1] and known to be positive for cytokeratin 20, a marker of colon cancer [5]. However, our case presented an unusual recurrence of adenocarcinoma involving the ureter, the renal pelvis, and the nephrostomy tract. Interestingly, all adenocarcinomas including anastomotic site tumors were positive for cytokeratin 7 but negative for cytokeratin 20 and *β*-catenin nuclear accumulation, which is uncommon for colon cancers. These findings suggest that anastomotic site tumors of our case might have originated from urothelium at the anastomotic site rather than colonic mucosa.

Multiplicity of tumors in the upper urinary tract may be explained by primary multicentric development of urothelial adenocarcinoma or tumor implantation of the anastomotic adenocarcinoma. Tumor implantation may be more likely in this case, since multiple cancerous lesions were found in the ureter, renal pelvis, and nephrostomy tract, with all the lesions showing indistinguishable histological appearance and identical immunohistochemistry, although primary multicentric adenocarcinoma of urothelium origin has been reported. [6].

Anastomotic site adenocarcinoma of the present case is potentially of urothelial origin because of unusual clinical manifestation and immunohistochemistry as a colon cancer.

## Abbreviations

CT: Computed tomography.
